# MAGE-A4, NY-ESO-1 and SAGE mRNA expression rates and co-expression relationships in solid tumours

**DOI:** 10.1186/s12885-020-07098-4

**Published:** 2020-06-29

**Authors:** Mikiya Ishihara, Shinichi Kageyama, Yoshihiro Miyahara, Takeshi Ishikawa, Shugo Ueda, Norihito Soga, Hiroaki Naota, Katsumi Mukai, Naozumi Harada, Hiroaki Ikeda, Hiroshi Shiku

**Affiliations:** 1 Cancer Center, Mie University Hospital, 2-174 Edobashi, Tsu, Mie 514-8507 Japan; 2 Department of Immuno-Gene Therapy, Mie University Graduate School of Medicine, 2-174 Edobashi, Tsu, Mie 514-8507 Japan; 3 Department of Personalized Cancer Immunotherapy, Mie University Graduate School of Medicine, 1577 Kurimamachiya-cho, Tsu, Mie 514-8507 Japan; 4 Department of Gastroenterology and Hepatology, Kyoto Prefectural University of Medicine, Kawaramachi-Hirokoji, Kajii-cho, Kamigyo-ku, Kyoto, 602-8566 Japan; 5 Department of Gastroenterological Surgery and Oncology, Kitano Hospital, The Tazuke Kofukai Medical Research Institute, 2-4-20 Ohgimachi, Kita-ku, Osaka, 530-8480 Japan; 6 Department of Urology, Aichi Cancer Center Hospital, 1-1 Kanokoden, Chikusa-ku, Nagoya, Aichi 464-8681 Japan; 7 Department of Gastroenterology, Matsusaka Chuo General Hospital, 102 Kobou, Kawai-machi, Matsusaka, Mie 515-8566 Japan; 8 Department of Gastroenterology, Suzuka General Hospital, 1275-53, Yamanohana, Yasuzuka-cho, Suzuka, Mie 513-8630 Japan; 9 United Immunity, Co., Ltd, Room 220, Mie University Campus Incubator, 1577 Kurimamachiya-cho, Tsu, Mie 514-8507 Japan; 10 Department of Oncology, Nagasaki University Graduate School of Biomedical Sciences, 1-12-4 Sakamoto, Nagasaki, Nagasaki 852-8523 Japan

**Keywords:** MAGE-A4, NY-ESO-1, qRT-PCR, SAGE, Solid tumour

## Abstract

**Background:**

Cancer testis (CT) antigens are promising targets for cancer immunotherapies such as cancer vaccines and genetically modified adoptive T cell therapy. In this study, we evaluated the expression of three CT antigens, melanoma-associated antigen A4 (MAGE-A4), New York oesophageal squamous cell carcinoma 1 (NY-ESO-1) and sarcoma antigen gene (SAGE).

**Methods:**

MAGE-A4, NY-ESO-1 and/or SAGE antigen expression in tumour samples was evaluated by quantitative real-time polymerase chain reaction (qRT-PCR). Informed consent was obtained from individuals prior to study enrolment.

**Results:**

In total, 585 samples in 21 tumour types were evaluated between June 2009 and March 2018. The positive expression rates of these CT antigens were as follows: MAGE-A4, 34.6% (range, 30.7–38.7); NY-ESO-1, 21.0% (range, 17.2–25.1); and SAGE, 21.8% (range, 18.5–25.4). The MAGE-A4 antigen was expressed in 54.9% of oesophageal cancers, 37.5% of head and neck cancers, 35.0% of gastric cancers and 34.2% of ovarian cancers; the NY-ESO-1 antigen was expressed in 28.6% of lung cancers, 25.3% of oesophageal cancers and 22.6% of ovarian cancers; and the SAGE antigen was expressed in 35.3% of prostate cancers, 32.9% of oesophageal cancers and 26.3% of ovarian cancers. The most common tumour type in this study was oesophageal cancer. MAGE-A4, NY-ESO-1 and SAGE antigen expression were assessed in 214 oesophageal cancer samples, among which 24 (11.2%) were triple-positive, 58 (27.1%) were positive for any two, 59 (27.6%) were positive for any one, and 73 (34.1%) were triple negative.

**Conclusions:**

Oesophageal cancer exhibited a relatively high rate of CT antigen mRNA expression positivity.

## Background

Cancer testis (CT) antigens are anticipated to be optimal targets for cancer immunotherapy because their expression is limited to the testis and placenta in normal tissue [1]. Since T. Boon et al. reported that melanoma-associated antigen (MAGE), a CT antigen, was recognized by T cells [2], many researchers have studied the potential of CT antigens as cancer immunotherapy targets [3]. Not only immune checkpoint inhibitors but also genetically modified T cell therapies, such as chimeric antigen receptor (CAR) and T cell receptor (TCR)-engineered T cell therapies, have been developed in this era of cancer immunotherapy [4–6]. CT antigens are anticipated to be target proteins for genetically modified T cell therapy.

MAGE-A4 [7, 8], New York oesophageal squamous cell carcinoma 1 (NY-ESO-1) [9–11] and sarcoma antigen gene (SAGE) [12] are CT antigens. Our group studied MAGE-A4- and SAGE-derived T cell epitopes [13] and conducted clinical trials using a cancer vaccine and/or TCR-engineered T cells targeting MAGE-A4- or NY-ESO-1-expressing tumours [14–19]. Before patients were enrolled in those clinical trials, CT antigen expression in tumour samples obtained from the patients was assessed as another clinical study, and we report the results here.

## Methods

### MAGE-A4, NY-ESO-1 and SAGE expression

RNA extraction was performed as described previously [20]. In brief, total RNA was extracted from frozen tissue samples, and complementary DNA (cDNA) was then prepared using a QuantiTect Reverse Transcription kit (Qiagen, Hilden, Germany). qRT-PCR was routinely performed. The sequences of the primers and probes used in our study were as follows: MAGE-A4, F: 5′-GCAGTAATCCTGCGCGCTAT-3′ and R: 5′-CATTGACCCTGACCACATGCT-3′; probe: 5′-FAM-CTCTGGCTGAAACCA-MGB-3′. NY-ESO-1, F: 5′-GGCTGAATGGATGCTGCAGA-3′ and R: 5′-CTGGAGACAGGAGCTGATGGA-3′; probe: 5′-FAM-TGTGTCCGGCAACATACTGACTATCCGA-TAMRA-3′. SAGE, F: 5′-TGTCATTCACGATATCCAGGAGG-3′ and R: 5′-GGTGGCATACAATGTCCTGTCAT-3′; probe: 5′-FAM-TGTGTCCGGCAACATACTGACTATCCGA-TAMRA-3′. Gene expression was evaluated as positive when the value exceeded 12.2 copies/10^4^ copies of glyceraldehyde-3-phosphate dehydrogenase (GAPDH) for MAGE-A4, 5.96 copies/10^4^ copies of GAPDH for NY-ESO-1 and 2.81 copies/10^4^ copies of GAPDH for SAGE. These cut-off values were determined as the means ±2 standard deviations (SDs) of the expression levels in the corresponding normal samples.

### Statistical analysis

Pearson’s chi-squared test of independence was used to evaluate associations between 2 variables. *P-*values of less than 0.05 were considered statistically significant. Calculations were performed with SPSS Statistics version 25 (IBM Japan, Ltd., Tokyo, Japan).

## Results

### CT antigen mRNA expression in tumours

Five hundred and 85 samples were collected and evaluated for MAGE-A4, NY-ESO-1 and/or SAGE expression between June 2009 and March 2018. The expression rates of MAGE-A4, NY-ESO-1 and SAGE were 34.6, 21.0 and 21.8%, respectively (Table 1).

**Table 1:** Rates of CT antigen mRNA expression

	Positive	Negative	Not Evaluable	Total Evaluated	Positive Rate (range)
MAGE-A4	199	376	10	585	34.6% (30.7–38.7)
NY-ESO-1	92	347	8	447	21.0% (17.2–25.1)
SAGE	125	449	7	581	21.8% (18.5–25.4)

Positive rate = 100 × (Positive)/(Positive + Negative).

Twenty-one tumour types were included in this study. The tumour types for which 5 or more samples were evaluated are listed in Tables 2-4. MAGE-A4 expression was evaluable in 575 samples. The MAGE-A4 expression rate was high in oesophageal cancer (54.9%), head and neck cancer (37.5%), gastric cancer (35.0%) and ovarian cancer (34.2%) (Table 2).

**Table 2:** MAGE-A4 expression in each tumour type

Type	Positive	Negative	Not Evaluable	Total Evaluated	Positive Rate (%)
Head and Neck	27	45	1	73	37.5
Oesophageal	124	102	6	232	54.9
Gastric	7	13	0	20	35.0
Colorectal	4	19	0	23	17.4
Lung	12	55	0	67	17.9
Ovarian	13	25	0	38	34.2
Endometrial	5	21	0	26	19.2
Cervical	2	18	1	21	10.0
Renal	0	45	0	45	0.0
Prostate	1	16	0	17	5.9

Positive rate = 100 × (Positive)/(Positive + Negative).

**Table 3:** NY-ESO-1 expression in each tumour type

Type	Positive	Negative	Not Evaluable	Total	Positive Rate (%)
Head and Neck	10	55	1	66	15.4
Oesophageal	55	162	4	221	25.3
Gastric	0	5	0	5	0.0
Colorectal	3	9	0	12	25.0
Lung	6	15	0	21	28.6
Ovarian	7	24	0	31	22.6
Endometrial	3	5	0	8	37.5
Cervical	0	3	1	4	0.0
Renal	3	42	0	45	6.7
Prostate	2	15	0	17	11.8

Positive rate = 100 × (Positive)/(Positive + Negative).

**Table 4:** SAGE expression in each tumour type

Type	Positive	Negative	Not Evaluable	Total Evaluated	Positive Rate (%)
Head and Neck	10	62	1	73	13.9
Oesophageal	74	151	3	228	32.9
Gastric	3	17	0	20	15.0
Colorectal	0	23	0	23	0.0
Lung	11	56	0	67	16.4
Ovarian	10	28	0	38	26.3
Endometrial	6	20	0	26	23.1
Cervical	1	19	1	21	5.0
Renal	2	43	0	45	4.4
Prostate	6	11	0	17	35.3

Positive rate = 100 × (Positive)/(Positive + Negative).

NY-ESO-1 was evaluable in 439 samples. The NY-ESO-1 expression rate was high in lung cancer (28.6%), oesophageal cancer (25.3%) and ovarian cancer (22.6%) (Table 3). Although the number of evaluable samples was limited, colorectal cancer and endometrial cancer also exhibited relatively high NY-ESO-1 expression rates.

SAGE was evaluable in 574 samples. The SAGE expression rate was high in prostate cancer (35.3%), oesophageal cancer (32.9%), ovarian cancer (26.3%) and endometrial cancer (23.1%) (Table 4).

The tumour types for which 4 or fewer samples were evaluated, which are not listed in the tables, were as follows: thyroid, small intestine, biliary tract, pancreatic, mesothelial, breast, urothelial, sarcoma, skin, multiple myeloma and unknown primary.

### Co-expression of CT antigens

**Fig. 1:**
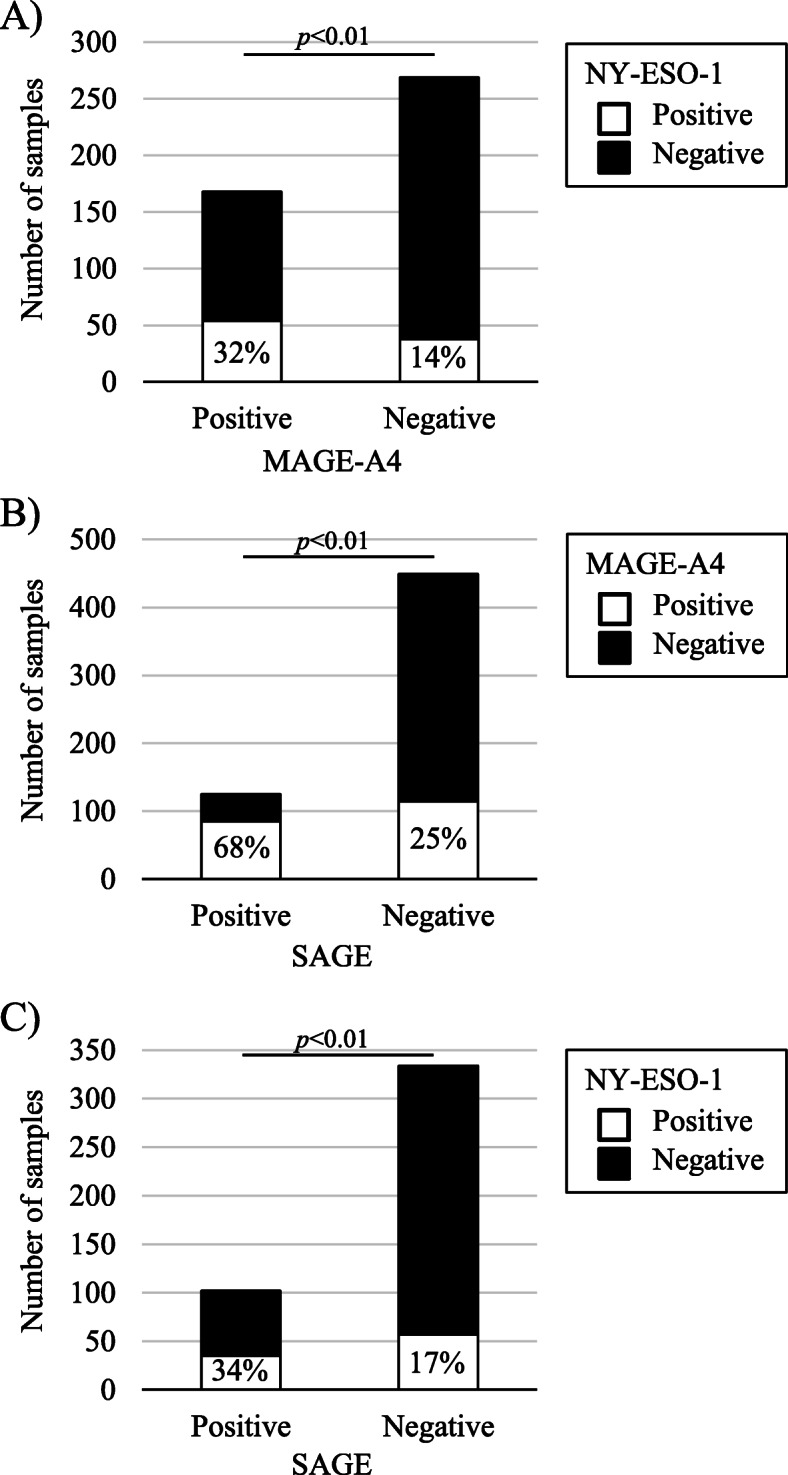
Relationship among MAGE-A4, NY-ESO-1 and SAGE mRNA expression. Pearson’s chi-squared test of independence was used for evaluation. There was a relationship among MAGE-A4, NY-ESO-1 and SAGE expression (all *p* < 0.01)

**Fig. 2:**
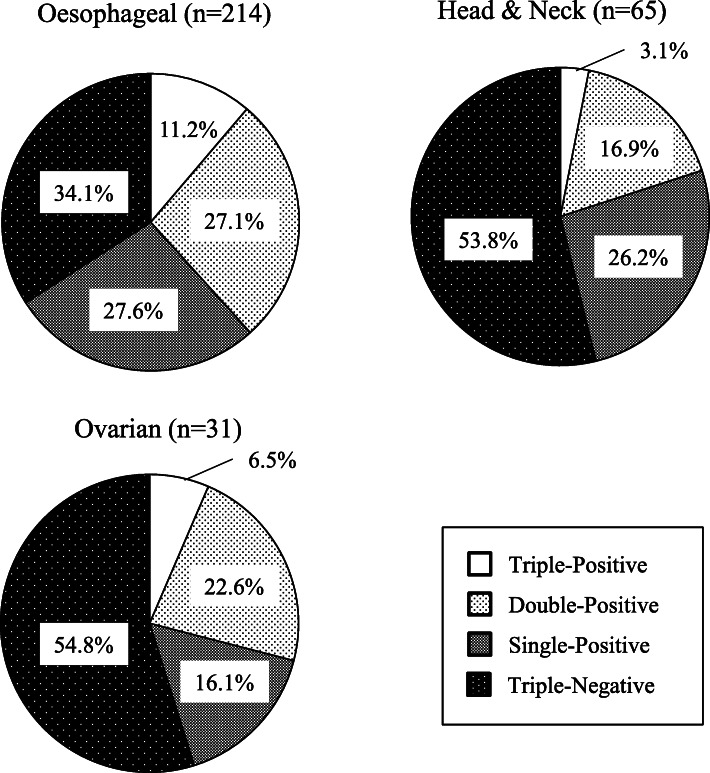
Co-expression of CT antigens in tumours. All 3 antigens were assessed in 436 patients. Among 21 tumour types, 3 with a high CT antigen expression rate were selected

MAGE-A4, NY-ESO-1 and SAGE mRNA expression levels demonstrated positive relationships (Fig. 1). To exclude the influence of oesophageal cancer, which accounted for approximately half of the assessed samples, we divided the samples into oesophageal cancer and other cancers for analysis. In the non-oesophageal tumour types, significant correlations in CT antigen co-expression, except for NY-ESO-1 and SAGE co-expression, were identified (Additional Fig. 1). All 3 CT antigens were assessed in 436 samples, and three tumour types with high CT antigen expression rates are shown in Fig. 2. In oesophageal cancer, 65.9% of tumours were positive for at least one CT antigen, and 38.3% expressed 2 or 3 CT antigens. Among these CT antigen-positive tumour types, the median copy numbers of MAGE-A4 and SAGE in oesophageal cancer were higher than those in the other 2 tumour types (Additional Table 1).

## Discussion

In this study, CT antigen expression was assessed in 585 tumour samples by quantitative real-time polymerase chain reaction (qRT-PCR). Among these tumour samples, 20–30% exhibited MAGE-A4, NY-ESO-1 and/or SAGE expression. The MAGE-A, NY-ESO-1 and SAGE expression rates in this study were comparable to those reported previously [11, 12, 21–27]. Among the 585 tumour samples, 214 oesophageal cancer samples were evaluable for the expression of all 3 CT antigens. This evaluation revealed a high CT antigen co-expression rate in oesophageal cancer.

CT antigens are promising targets for cancer immunotherapy. For example, NY-ESO-1-specific TCR-engineered T cell therapy has shown promising antitumour responses in clinical trials [17, 28, 29]. Our data will be useful for considering the next cancer immunotherapy target. In addition, CT antigen expression and/or anti-CT antigen antibodies may have potential as biomarkers. Indeed, some reports have examined the impact of these factors on survival [30–32]. The impact of CT antigens on survival is controversial, possibly because of differences in tumour type, tumour stage and/or tumour burden. Non-targeted antigen-specific T cell responses and/or antibody production, known as antigen spreading, often occurs during cancer immunotherapy. As antigen spreading may be helpful in guiding the response to immunotherapy early in the treatment course [33, 34], further assessments of CT antigens as prognostic factors are expected.

The reason that CT antigen co-expression is high in oesophageal cancer has not yet been clarified. The expression of MAGE-A and NY-ESO-1 is mediated by demethylation of their promoters [35–38]. As MAGE-A, NY-ESO-1 and SAGE antigens are located in the q28 region on the X chromosome, demethylation of common promoters or those that are located nearby may occur. In this study, a high CT antigen expression rate was observed in oesophageal cancer. In general, CT antigen expression seemed to increase as the tumour progressed. However, one report suggested that CT antigen expression was high in low-grade oesophageal cancer [24]. The oesophagus may be more susceptible to demethylation than other organs.

We identified 5 reports about the co-expression of CT antigens in oesophageal cancer in international journals. Among these 5 reports, 4 assessed CT antigen expression by immunohistochemistry (IHC) [21, 22, 24, 25] and 1 assessed it by PCR [23]. IHC can be performed on formalin-fixed, paraffin-embedded samples, enabling us to study a large number of samples retrospectively. However, CT antigen expression assessment via IHC can lack confidence. The anti-NY-ESO-1 antibody D8.38 recognizes not only NY-ESO-1 but also L antigen family member 1 (LAGE-1), which is also called NY-ESO-2 [21]. In addition, 57B, an anti-MAGE antibody often used to assess MAGE expression by IHC, cannot distinguish between members of the MAGE-A subfamily [21, 39]. Forghanifard et al. [23] assessed CT antigen expression in oesophageal squamous cell carcinoma by PCR and reported a positive relationship between MAGE-A4 and NY-ESO-1 and between MAGE-A4 and LAGE-1. However, their report showed a MAGE-A4 expression rate of 90.2% in oesophageal squamous cell cancer. Although the assessment method differed (IHC vs PCR), the positive rate of MAGE-A4 expression was excessively high compared with that indicated in previous reports. The positive rates of MAGE-A4, NY-ESO-1 and SAGE expression in our study were comparable to those reported previously. In addition, the number of samples assessed for CT antigen co-expression in oesophageal cancer was larger than that in the study reported by Forghanifard et al. [23] (214 samples vs 41 samples). MAGE-A4, NY-ESO-1 and SAGE mRNA expression in normal tissue is shown in Additional Fig. 2a-c. As illustrated in Additional Fig.2b, NY-ESO-1 was positive in normal prostate. Lethe et al. previously reported lack of NY-ESO-1 mRNA expression in normal prostate [40]. The frequency of NY-ESO-1 mRNA expression in prostate cancer was 11.8% in our study. Latent prostate cancer might be involved.

This study has some limitations. First, all samples were assessed in a single institute. This strategy assured consistent methods and yielded reliable results, but the universality of our assessment was not confirmed. Second, details of histological types were not collected, because this study aimed to assess CT expression in tumour samples obtained from patients who hoped to enrol in clinical studies of CT antigen-targeting cancer immunotherapies. However, histological differences may affect the rate of CT antigen expression even in cancers of the same primary organ. For example, MAGE-A4 was more frequently expressed in lung squamous cell carcinoma than in lung adenocarcinoma [41], and the NY-ESO-1 expression rates in synovial sarcoma and myxoid round cell liposarcoma were higher than those in other types of soft tissue sarcoma [42]. Among patients enrolled in this study, oesophageal cancer was the most common type. In Japan, oesophageal squamous cell carcinoma accounts for approximately 90% of oesophageal cancers, and oesophageal adenocarcinoma is rare [43]. Thus, the CT antigen expression rate in oesophageal carcinoma in this study could be interpreted to reflect mainly oesophageal squamous cell carcinoma. Third, qRT-PCR analyses do not always reflect the CT antigen expression status in the whole tumour, because tumours often exhibit heterogeneity. Moreover, importantly, qRT-PCR analyses cannot confirm protein production in tumours, because qRT-PCR assesses only mRNA expression. Both IHC and qRT-PCR were assessed in 41 of MAGE-A4 mRNA-examined samples and 20 of NY-ESO-1 mRNA-examined samples. In IHC analyses, MAGE-A4 positivity was defined as MCV-1 positivity and MCV-4 positivity [16], NY-ESO-1 positivity was defined as E978 positivity [15, 19]. SAGE IHC was not assessed because of a lack of an appropriate antibody. IHC sensitivity and specificity were 64 and 75% for MAGE-A4 mRNA assessment, and 60 and 93% for NY-ESO-1 mRNA assessment, respectively (Additional Table 2); for approximately 40% of mRNA expression-positive tumour samples, protein production could not be confirmed. Despite these limitations, the large number of tumour samples, especially oesophageal cancer samples, is a strength of this study.

## Conclusions

This study assessed MAGE-A4, NY-ESO-1 and/or SAGE antigen expression in 585 tumour samples. Oesophageal cancer exhibited a high rate of CT antigen mRNA expression and a high rate of CT antigen mRNA co-expression.

## Supplementary information

**Additional file 1 **Figure 1**.** Relationship among MAGE-A4, NY-ESO-1 and SAGE mRNA expression in oesophageal cancer and other cancer types. Pearson’s chi-squared test of independence was used for evaluation. In oesophageal cancer, there was a relationship among MAGE-A4, NY-ESO-1 and SAGE expression (A-C, left; all *p* < 0.01). In other cancer types, there was a relationship between MAGE-A4 and NY-ESO-1 expression (*p* < 0.01) (A, right) and between MAGE-A4 and SAGE expression (*p* < 0.01) (B, right) but not between NY-ESO-1 and SAGE expression (*p* = 0.14) (C, right).

**Additional file 2 **Figure 2**.** CT antigen mRNA expression in normal tissue. mRNA expression of MAGE-A4 (A), NY-ESO-1 (B) and SAGE (C) in normal tissue was shown. First Choice™ Human Total RNA Survey Panel®, Human Breast Total RNA®, Human Lymph node Total RNA®, Human Testicle Total RNA® and Human Uterus Total RNA® (Ambion KK, Tokyo, Japan) were used.

**Additional file 3 **Table 1**.** Median levels of CT antigen mRNAs in each CT antigen-positive tumour type.

**Additional file 4 **Table 2**.** MAGE-A4 and NY-ESO-1 IHC analyses of mRNA-assessed tumour samples.

## Data Availability

The datasets generated and/or analysed in the present study are not publicly available to protect patient information in the study database, but they are available from the corresponding author upon request.

## Abbreviations

CAR: Chimeric antigen receptor
CT: Cancer testis
GAPDH: Glyceraldehyde-3-phosphate dehydrogenase
IHC: Immunohistochemistry
LAGE-1: L antigen family member 1
MAGE: Melanoma-associated antigen
NY-ESO-1: New York oesophageal squamous cell carcinoma 1
qRT-PCR: Quantitative real-time polymerase chain reaction
SAGE: Sarcoma antigen gene
SD: Standard deviation
TCR: T cell receptor
