# Supplementary material for: MAGE-A4, NY-ESO-1 and SAGE mRNA expression rates and co-expression relationships in solid tumours
Source: BMC Cancer. 2020 Jun 29;20:606. doi: 10.1186/s12885-020-07098-4 (PMC7325278; doi:10.1186/s12885-020-07098-4)
Supplement: Supplementary file 1 — Additional file 1 Figure 1. Relationship among MAGE-A4, NY-ESO-1 and SAGE mRNA expression in oesophageal cancer and other cancer types. Pearson’s chi-squared test of independence was used for evaluation. In oesophageal cancer, there was a relationship among MAGE-A4, NY-ESO-1 and SAGE expression (A-C, left; all p < 0.01). In other cancer types, there was a relationship between MAGE-A4 and NY-ESO-1 expression (p < 0.01) (A, right) and between MAGE-A4 and SAGE expression (p < 0.01) (B, right) but not between NY-ESO-1 and SAGE expression (p = 0.14) (C, right). [file 12885_2020_7098_MOESM1_ESM.pptx]

## Slide 1
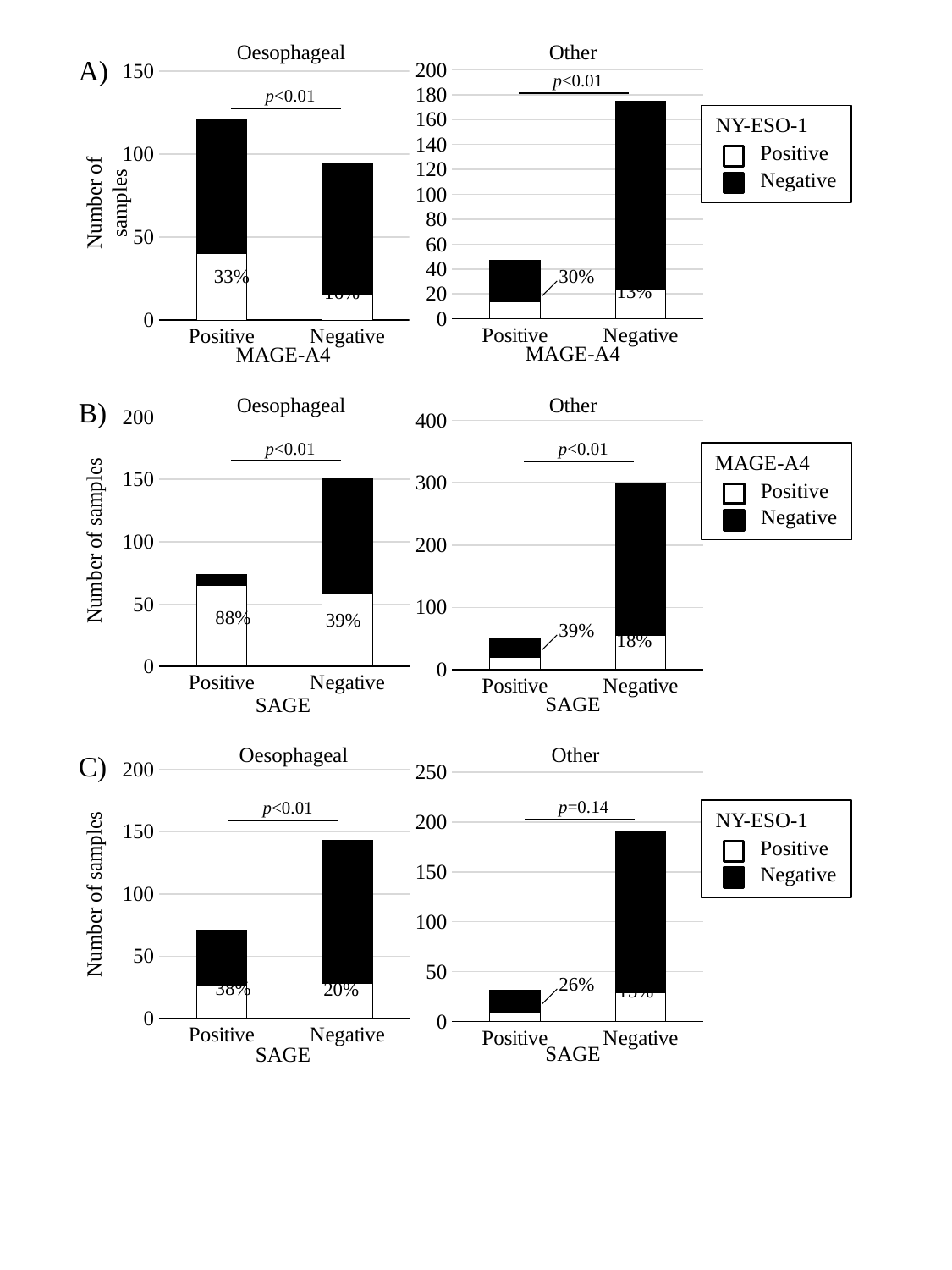

Oesophageal
Other
A)
### Chart
| Category | Positive | Negative |
|---|---|---|
| Positive | 14.0 | 33.0 |
| Negative | 23.0 | 152.0 |
### Chart
| Category | Positive | Negative |
|---|---|---|
| Positive | 40.0 | 81.0 |
| Negative | 15.0 | 79.0 |p<0.01
p<0.01
NY-ESO-1
Positive
Negative
Number of samples
33%
30%
13%
16%
MAGE-A4
MAGE-A4
Oesophageal
Other
B)
### Chart
| Category | Positive | Negative |
|---|---|---|
| Positive | 65.0 | 9.0 |
| Negative | 59.0 | 92.0 |
### Chart
| Category | Positive | Negative |
|---|---|---|
| Positive | 20.0 | 31.0 |
| Negative | 55.0 | 243.0 |p<0.01
p<0.01
MAGE-A4
Positive
Negative
Number of samples
88%
39%
39%
18%
SAGE
SAGE
Oesophageal
Other
C)
### Chart
| Category | Positive | Negative |
|---|---|---|
| Positive | 27.0 | 44.0 |
| Negative | 28.0 | 115.0 |
### Chart
| Category | Positive | Negative |
|---|---|---|
| Positive | 8.0 | 23.0 |
| Negative | 29.0 | 162.0 |p=0.14
p<0.01
NY-ESO-1
Positive
Negative
Number of samples
26%
38%
20%
15%
SAGE
SAGE
