# Supplementary material for: MAGE-A4, NY-ESO-1 and SAGE mRNA expression rates and co-expression relationships in solid tumours
Source: BMC Cancer. 2020 Jun 29;20:606. doi: 10.1186/s12885-020-07098-4 (PMC7325278; doi:10.1186/s12885-020-07098-4)
Supplement: Supplementary file 2 — Additional file 2 Figure 2. CT antigen mRNA expression in normal tissue. mRNA expression of MAGE-A4 (A), NY-ESO-1 (B) and SAGE (C) in normal tissue was shown. First Choice™ Human Total RNA Survey Panel®, Human Breast Total RNA®, Human Lymph node Total RNA®, Human Testicle Total RNA® and Human Uterus Total RNA® (Ambion KK, Tokyo, Japan) were used. [file 12885_2020_7098_MOESM2_ESM.pptx]

## Slide 1
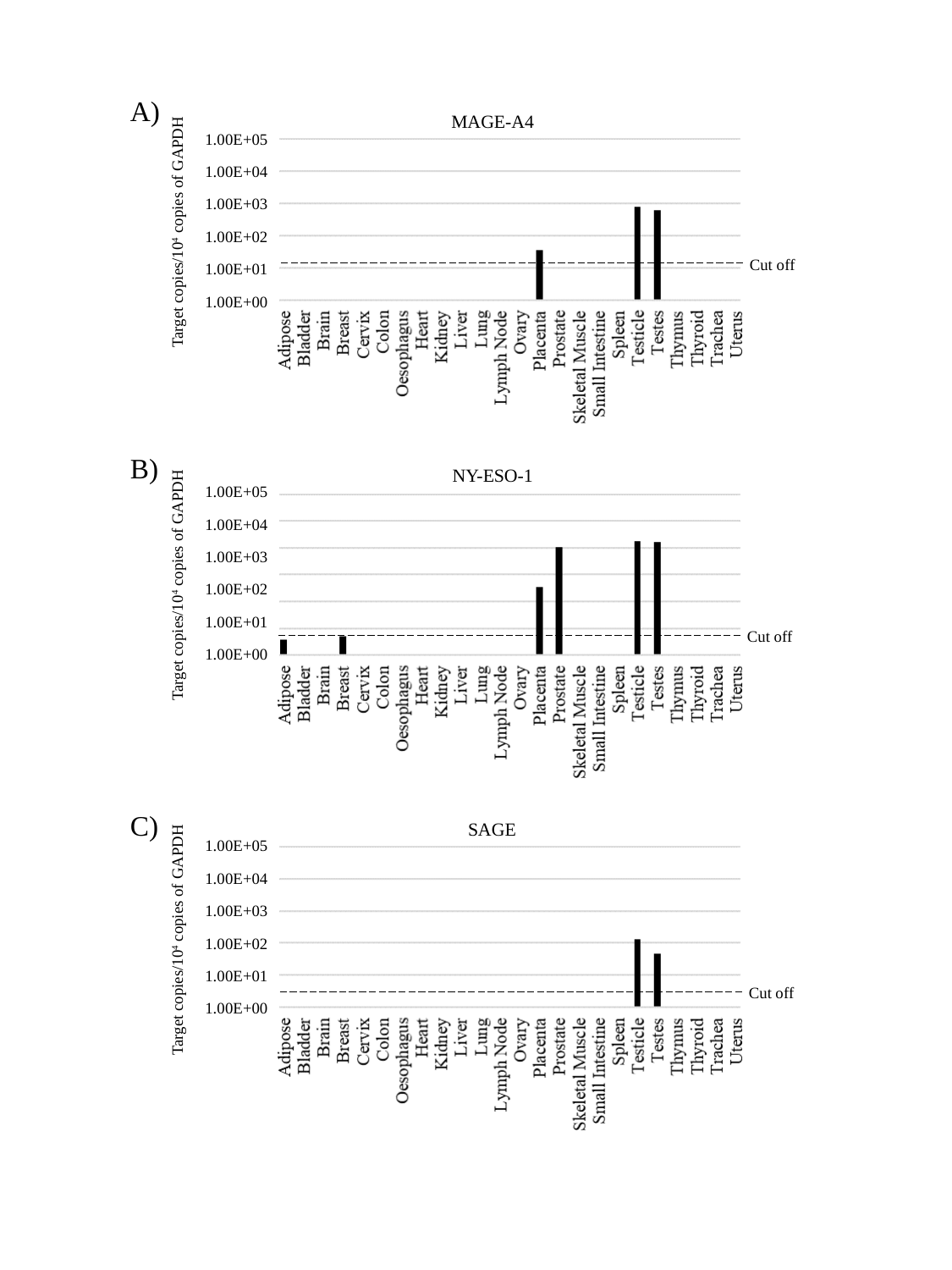

A)
MAGE-A4
Target copies/104 copies of GAPDH
1.00E+05
1.00E+04
1.00E+03
1.00E+02
1.00E+01
1.00E+00
Cut off
B)
NY-ESO-1
Target copies/104 copies of GAPDH
1.00E+05
1.00E+04
1.00E+03
1.00E+02
1.00E+01
1.00E+00
Cut off
C)
SAGE
Target copies/104 copies of GAPDH
1.00E+05
1.00E+04
1.00E+03
1.00E+02
1.00E+01
1.00E+00
Cut off
