# Supplementary material for: MAGE-A4, NY-ESO-1 and SAGE mRNA expression rates and co-expression relationships in solid tumours
Source: BMC Cancer. 2020 Jun 29;20:606. doi: 10.1186/s12885-020-07098-4 (PMC7325278; doi:10.1186/s12885-020-07098-4)
Supplement: Supplementary file 3 — Additional file 3 Table 1. Median levels of CT antigen mRNAs in each CT antigen-positive tumour type. [file 12885_2020_7098_MOESM3_ESM.docx]

**Additional Table 1.** Median levels of CT antigen mRNAs in each CT antigen-positive tumour type.

|  | **All** | **Head and Neck** | **Oesophageal** | **Ovarian** |
| --- | --- | --- | --- | --- |
| **MAGE-A4** | 445.0 | 471.5 | 633.4 | 133.0 |
| **NY-ESO-1** | 47.2 | 127.3 | 51.0 | 12.9 |
| **SAGE** | 42.0 | 33.9 | 70.1 | 34.6 |

(copies/10^4^ GAPDH copies)
