# Supplementary material for: MAGE-A4, NY-ESO-1 and SAGE mRNA expression rates and co-expression relationships in solid tumours
Source: BMC Cancer. 2020 Jun 29;20:606. doi: 10.1186/s12885-020-07098-4 (PMC7325278; doi:10.1186/s12885-020-07098-4)
Supplement: Supplementary file 4 — Additional file 4 Table 2. MAGE-A4 and NY-ESO-1 IHC analyses of mRNA-assessed tumour samples. [file 12885_2020_7098_MOESM4_ESM.docx]

**Additional Table 2.** MAGE-A4 and NY-ESO-1 IHC analyses of mRNA-assessed tumour samples.

| **MAGE-A4** | | **qRT-PCR** | |
| --- | --- | --- | --- |
|  |  | Positive | Negative |
| **IHC** | Positive | 21 | 2 |
|  | Negative | 12 | 6 |

IHC: sensitivity 64% (21/33), specificity 75% (6/8).

| **NY-ESO-1** | | **qRT-PCR** | |
| --- | --- | --- | --- |
|  |  | Positive | Negative |
| **IHC** | Positive | 3 | 1 |
|  | Negative | 2 | 14 |

IHC: sensitivity 60% (3/5), specificity 93% (14/15).
